# Virtual Reality Applications in Neurorehabilitation: Current Panorama and Challenges

**DOI:** 10.3390/brainsci13050819

**Published:** 2023-05-18

**Authors:** Francisco Nieto-Escamez, Irene Cortés-Pérez, Esteban Obrero-Gaitán, Augusto Fusco

**Affiliations:** 1 Department of Psychology, University of Almeria, 04120 Almeria, Spain; pnieto@ual.es; 2 Center for Neuropsychological Assessment and Rehabilitation (CERNEP), 04120 Almeria, Spain; 3 Department of Health Sciences, University of Jaen, Paraje Las Lagunillas s/n, 23071 Jaen, Spain; eobrero@ujaen.es; 4 UOC Neuroriabilitazione ad Alta Intensità, Fondazione Policlinico Universitario A. Gemelli IRCCS, 00168 Rome, Italy

Central Nervous System Diseases are a leading cause of disability worldwide, posing significant social and economic burdens for patients, their families, caregivers, and society as a whole. In an ageing population, these diseases have a substantial impact and are expected to continue to increase [1]. Consequently, effective clinical management and rehabilitation treatments are necessary to reduce the associated disabilities.

In the present special issue, we will try to gather evidence on whether and how Virtual Reality (VR)-based interventions may help to mitigate impairments, increase engagement in activities, and improve quality of life. VR represents a paradigm shift in rehabilitation due to its capacity to provide meaningful and realistic experiences that adapt learning principles to the intervention. Learning is improved when tasks are relevant and repetitive, and when task difficulty is gradually increased [2]. VR permits the customization of the number of stimuli and the difficulty of tasks to the patient’s requirements and abilities, maintaining stimulus control and consistency, and providing real-time and goal-directed feedback [3].

Due to research progress and the decreasing cost of virtual technology, VR has steadily become a valuable tool for assessment and intervention in clinical rehabilitation for several conditions [4]. VR has been found to be effective in the rehabilitation of upper limb extremities and balance in stroke patients [5], as well as in improving functional mobility and balance in older adults [6], children with cerebral palsy [7] and in individuals with Parkinson’s disease [8] and multiple sclerosis [9]. Furthermore, successful applications of VR-based therapies have been demonstrated for the management of acute [10] and chronic [11] pain. Finally, it has been used to promote well-being in people with dementia [12], and enhance cognitive function in subjects with multiple sclerosis [13], and in older adults with mild cognitive impairment [14].

Nevertheless, the literature has reported conflicting evidence, from no significant improvement when VR is compared to conventional therapies, to an enhancement of rehabilitation outcomes when it is used alone or as an additional treatment. Recently, Voinescu et al. [15] reviewed the evidence from meta-analyses on the efficacy of VR-based therapies in many physical and cognitive domains of several neurological conditions, generally reporting low- or very low-quality evidence supporting the effectiveness of VR-based interventions.

Many factors should be taken into account to objectively assess the efficacy of VR in neurorehabilitation. A large body of literature supports the importance of technology components underpinning VR treatments, as well as the role of personalized VR interventions as an effective treatment. In general, personalized VR systems are recommended over commercially available VR systems (e.g., Nintendo Wii, Microsoft Kinect), particularly for upper limb extremities, body function, and activity [16]. Consequently, bespoke VR systems adhere more closely to rehabilitation principles by adapting to user needs and skills, providing feedback, practicing the affected body part, and increasing difficulty [17]. Nevertheless, commercial VR devices are generally more affordable and widely accessible, implementing its use in healthcare facilities with limited budgets, or even at home and allowing for greater availability in delivering these interventions to a larger population. The choice between commercial VR devices and customized systems should be based on the specific needs of the patient and target of the neurorehabilitation program, taking into consideration factors such as budget, accessibility, ease of use, and desired outcomes. It would be worth developing comparative studies among different technologies including cost-effectiveness analysis, along with the usual outcome measures and acceptability, as well as the sustainability and durability of the outcomes.

A key factor to differentiate between different VR solutions is the level of immersion and presence they provide. According to their technology, VR systems are often categorized as immersive, semi-immersive, or non-immersive, although these boundaries are sometimes nuanced [18]. In general, it is widely agreed that technological elements of VR systems that make the experience more immersive (e.g., including motion tracking and interaction technologies, a larger range of vision, and stereoscopy) boost a subjective state of awareness that describes the amount to which people feel “there” in VR called presence [19]. Adequate immersion and presence enable users to behave in VR as they would in real life [20] and may contribute to the successful transfer of trained skills [21]. Nevertheless, healthcare professionals should consider each patient’s capabilities, preferences, and rehabilitation goals when selecting the appropriate level of immersion, and closely monitor and adjust its level as needed during the rehabilitation process [22]. Although some studies have suggested that higher levels of immersion produce more realistic training experiences, and potentially lead to more effective rehabilitation, many factors can influence VR-based neurorehabilitation outcomes. In addition, semi-immersive and non-immersive VR can also be effective in neurorehabilitation, depending on the targets and requirements of the rehabilitation program. Further research is needed to better understand the optimal level of immersion that can influence VR-based interventions’ effectiveness.

Another topic of our Special Issue is how VR-based interventions can be integrated with other strategies and technologies. For instance, comparing VR standalone interventions with VR interventions delivered in combination with conventional therapy, or combining VR with other solutions. These combinations can enhance the effectiveness of interventions by leveraging the benefits of different technologies. Some examples of technologies employed alongside VR include robotics, different kind of sensors, and brain–computer interfaces (BCI) [23]. The integration of these technologies has emerged as a cutting-edge approach to enhance rehabilitation outcomes, offering a dynamic and interactive environment that can be customized to the needs and abilities of individual patients, while robots provide assistance and feedback in real-time, enabling precise and targeted rehabilitation interventions [24]. Sensors, such as motion capture devices and physiological sensors, collect objective data on patients’ movements, muscle activity, and physiological responses, which can be used to tailor therapy programs and track progress [25,26]. In addition, BCIs allow patients to control robotic devices or interact with VR environments using brain signals, facilitating neurofeedback and neuroplasticity [27]. This integrated approach has shown promising results in improving motor function, cognitive function, and quality of life in patients with various conditions, such as stroke, spinal cord injury, and traumatic brain injury [24,27,28,29]. Nevertheless, although the alliance among these technologies shows promising results, the evidence is still evolving, and further research is required to establish standardized protocols and guidelines for their use. Researchers and clinicians must evaluate which factors ensure these technologies are properly tailored to each patient’s individual needs and abilities and develop protocols to ensure their safety. This will help to understand their potential benefits and limitations for improving rehabilitation outcomes.

VR-based neurorehabilitation should be considered from a multidisciplinary perspective. Clinical expertise is critical to tailor VR interventions. At the same time, feedback from experts in technology and engineering who contribute to VR systems’ design, development, and customization is also needed. Psychosocial and behavioral aspects that may impact patients’ engagement should be considered as well. Finally, a patient-centered approach requires input from patients, caregivers, and their families. This multidisciplinary perspective should be considered in the basic research and applied clinical studies for VR-based neurorehabilitation development.

With this Special Issue, we aim to provide new evidence regarding how VR can improve neurorehabilitation outcomes. Nevertheless, we are aware of some challenges regarding the current status of this field of research. In particular, the novelty of the topic and the heterogeneity of patient populations result in a lack of standardized protocols, guidelines, and measures for evaluating the effectiveness, safety, and usability of VR interventions. It will be worthwhile to provide studies that compare and synthesize research findings and help to establish evidence-based practices in the field. Along the same line, VR technologies are rapidly evolving, and there is a wide range of VR hardware, software, and platforms available in the commercial market, which can differ in terms of quality, capabilities, and costs. It is essential to analyze such variability in terms of standardization, reproducibility, generalizability of research findings, and cost-effectiveness. It is also essential to provide evidence about the effectiveness and sustainability of VR-based interventions over time. As a general recommendation, for the articles published in this Special Issue we would like a final section entitled “The Potential Impact of Virtual Reality on Clinical Practice” to be included in the submitted manuscripts. This will be helpful for neurorehabilitation professionals in their daily work providing clinical care to their patients.
